# One-Time Optimization of Advanced T Cell Culture Media Using a Machine Learning Pipeline

**DOI:** 10.3389/fbioe.2021.614324

**Published:** 2021-07-15

**Authors:** Paul Grzesik, Sebastian C. Warth

**Affiliations:** R&D Cell Culture Systems, CellGenix GmbH, Freiburg, Germany

**Keywords:** T cells, culture media design, machine learning, design of experiment, screening, cell culture, cell and gene therapy, donor variability

## Abstract

The growing application of cell and gene therapies in humans leads to a need for cell type-optimized culture media. Design of Experiments (DoE) is a successful and well known tool for the development and optimization of cell culture media for bioprocessing. When optimizing culture media for primary cells used in cell and gene therapy, traditional DoE approaches that depend on interpretable models will not always provide reliable predictions due to high donor variability. Here we present the implementation of a machine learning pipeline into the DoE-based design of cell culture media to optimize T cell cultures in one experimental step (one-time optimization). We applied a definitive screening design from the DoE toolbox to screen 12 major media components, resulting in 25 (2*k* + 1) media formulations. T cells purified from a set of four human donors were cultured for 6 days and cell viability on day 3 and cell expansion on day 6 were recorded as response variables. These data were used as a training set in the machine learning pipeline. In the first step, individual models were created for each donor, evaluated and selected for each response variable, resulting in eight final statistical models (*R*^2^ > 0.92, RMSE < 1.5). These statistical models were used to predict T cell viability and expansion for 10^5^ random *in silico*-generated media formulations for each donor in a grid search approach. With the aim of identifying similar formulations in all donors, the 40 best performing media formulations of each response variable were pooled from all donors (*n* = 320) and subjected to unsupervised clustering using the k-means algorithm. The median of each media component in each cluster was defined as the cluster media formulation. When these formulations were tested in a new set of donor cells, they not only showed a higher T cell expansion than the reference medium, but also precisely matched the average expansion predicted from the donor models of the training set. In summary, we have shown that the introduction of a machine learning pipeline resulted in a one-time optimized T cell culture medium and is advantageous when working with heterogeneous biological material.

## Introduction

In autologous cell therapy approaches, cells from a given patient are isolated, may be genetically modified to fulfill a therapeutic purpose and expanded in order to provide a sufficient dose of the cell product (Kazmi et al., 2009; June et al., 2018). In case of T cell therapy, the donor material is isolated from peripheral blood and consists of T cells and multiple other cell types. Even when T cells are purified by their common surface marker CD3, they differ in each donor in expression of other surface markers as well as in their metabolic and functional capacity (Mahnke et al., 2013; Klein Geltink et al., 2018). In the manufacturing process, these cells are typically activated by ligation of the T cell co-receptors CD3 and CD28 to trigger expansion of T cells and are cultured for several days in culture media supplemented with appropriate cytokines (Trickett and Kwan, 2003; Xu et al., 2014). In this process, an efficient and robust expansion of T cells from any donor regardless of the heterogeneity of cell populations is essential not only to meet the specifications of good manufacturing practice but also because timely manufacturing of the cell product can be critical to patient treatment (Gee, 2018). This can be achieved with an optimized cell culture medium formulation that supports the expansion of each donor’s T cells.

Cell culture media are complex mixtures of substances such as nutrients, salts, trace elements, buffers, hormones, carrier proteins, etc. (Yao and Asayama, 2017). For each cell type, the optimal composition and amounts of these components must be determined by suitable experimental setups.

For decades, the Design of Experiments (DoE) has been used for the development of cell culture media (Yao and Asayama, 2017). Sequential strategies, such as screening of several components followed by characterization of the relationships between the variables and finally their optimization, have proven to be particularly successful in bioprocessing (Castro et al., 1992; Kim et al., 1998; Liu et al., 2001). Recently, high-throughput technology in combination with exhaustive experimental designs has enabled rapid optimization of the medium for fed-batch cultures within a short time (Jordan et al., 2013; Rouiller et al., 2013; Bayer et al., 2020). While these strategies can be easily implemented using established cell lines such as Chinese hamster ovarian cells, the design of cell culture media for primary T cells is of greater challenge. The heterogeneous populations of cells purified from different human donors show a high degree of variability in cell expansion and viability, which leads to donor-dependent effect sizes of the screened media components and therefore makes sequential strategies with different donors in succession difficult.

**FIGURE 1:**
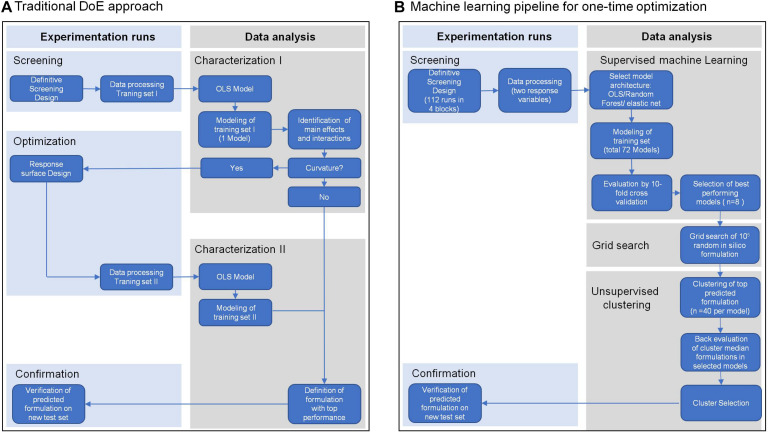
Overview of the traditional Design of Experiment (DoE) approach **(A)** and of a machine learning pipeline **(B)**. Both approaches include a screening step for data collection, in this case from expansion of T cells from four different healthy donors in a definitive screening design. Traditional DoE uses interpretable model architectures across all donors such as ordinary least squares regression (OLS) to select significant features (Characterization I). A second experimental step is applied to screen for optimal parameter levels (Optimization II). In a second modeling step using OLS the optimal parameter levels are determined for an optimal media formulation (Characterization II) that is experimentally confirmed (Confirmation). The machine learning pipeline **(B)** uses competitive machine learning algorithms to generate complex models for every response variable in every donor, which allow high prediction accuracy but are less interpretable (Supervised machine learning). After cross-validation these models are used to select the top 40 media formulations for every donor and every response variable from a random set of 10^5^ *in silico* media formulations (Grid search). The top 40 formulations of all donors and responses are clustered by formulation similarity and a cluster formulation was defined by the median component level of all formulations in a cluster (Unsupervised Clustering). Back evaluation of cluster formulations in the donor models for every response allows selection of the media formulation with the best response across all donors and responses, which again is experimentally confirmed (Confirmation).

To meet these challenges, we present in this study the extension of traditional experimental design with machine learning to optimize a cell culture medium for T cell expansion in one step. We applied a definitive screening design (Jones and Nachtsheim, 2011) with a minimum number of tested formulations, which allowed screening of a maximum number of different components in a given experimental system. In traditional workflows (Figure 1A) scientists focus on inference using interpretable model architectures such as ordinary least squares regression (OLS) to select significant features based on cell biological understanding. In contrast, we used competitive machine learning algorithms such as *elastic net regularized general linear models* (Zou and Hastie, 2005) and *random forest* (Breiman and Schapire, 2001). These complex model architechtures generate highly complex models which are less interpretable than traditional OLS models but have better prediction accuracy (Figure 1B). These algorithms were used to build individual high-performance statistical models, to predict cell expansion and viability of random *in silico* generated media formulations. We aimed to identify media compositions that encompass different media requirements of cells from individual donors. For this purpose, we pooled the top 40-predicted media formulations from each donor and used *k*-means clustering to identify clusters across donors with similar compositions. Using the median component level of each cluster we defined a cluster medium formulation that would potentially support expansion of cells from all donors. Finally, we demonstrated the enhanced performance of the selected cluster medium formulations in a confirmation experiment against other test and reference media for T cell expansion on a new test set of four different donors. The evaluation of model performances on the test set showed that our machine learning models were able to predict T cell expansion with higher precision than a single response linear regression model based on pooled data (traditional approach).

## Materials and Methods

### Cell Culture

CD3^+^ T cells were purified from healthy human donor blood preparations by negative magnetic bead isolation (EasySep^TM^ Human T Cell Isolation Kit, Stemcell Technologies) and cryopreserved. Thawed cells were activated using Dynabeads Human T-Activator CD3/CD28 (Thermo Fisher) at a ratio of 1:1 bead:cell in test media or reference media in the presence of IL-7 (10 ng/ml, CellGenix) and IL-15 (10 ng/ml, CellGenix). Cells were cultured at 37 °C in a humidified incubator at 5% CO_2_ in 96-well U bottom plates with two to three repeats per condition. On day 3 cells were splitted and reseeded with cytokine-containing media. To determine cell viability, cells were labeled with 7-Amino-Actinomycin D (7-AAD, BD-Pharmingen) and analyzed by flow cytometry. Cell count was determined using an Attune Nxt Flow Cytometer (Thermo Fisher).

### Culture Media

Test media were prepared from a common base medium with the 12 test components added at the appropriate amounts according to the DoE levels. To control for media preparation, a reference medium with defined expansion properties was prepared in parallel. All media were adjusted to pH = 7.2 and osmolality = 300 mOsm/Kg H_2_O.

### Data Processing and Modeling

All statistical analysis were carried out with the statistical software R v3.6.0 (R Core Team, 2020) and RStudio v1.1.463 (2009–2018 RStudio, Inc.). Data processing and visualization was carried out with package tidyverse v1.2.1. Design of Experiments was carried out with package daewr v1.1-7 and rsm v2.1.0. Modeling and feature selection was carried out with package caret (using the integrated ranger package v4.6-7, glmnet package v2.0.18 and MASS package v7.3-51.4). Cluster analysis was carried out with package cluster v2.1.0 and clustertend v1.4, visualization of multivariate data analysis was carried out with package factoextra v1.0.6.

As statistical tests in sections “Clustering and Selection of in silico Formulations”, “Evaluation of Selected Cluster Medium Formulation on a New Set of Donors”, and “Evaluation of Model Performance” an ANOVA followed by Tukey *post hoc* test at a significance level <0.05 was performed.

## Results

### Strategy Outline

We aimed to improve a proprietary base medium formulation for the expansion of primary human T cells. In contrast to a traditional optimization strategy based on sequential screening, characterization and optimization (sco) steps (Anderson, 2019) (Figure 1A), we carried out a one-time media optimization using a machine learning pipeline. We reasoned that cell viability at an early stage of cell culture might be influenced by an independent set of components than the T cell expansion at the end of culture, which could additionally contribute to overall expansion. Therefore, we selected T cell viability on day 3 and T cell expansion on day 6 as response variables. To account for the donor variability, we included cells from four human donors into the analysis as independent experimental blocks. This data served as our training set for the modeling steps. Our strategy consisted of following steps (Figure 1B) which are described in detail in sections “Data Collection,” “Data Modeling,” “Prediction of *In Silico* Formulations,” “Clustering and Selection of High-Performance *In Silico* Formulations,” and “Evaluation of Selected Cluster Medium Formulations on a New Set of Donors.”

### Data Collection

We selected 12 cell culture media components (c01–c12), which might impact the performance of the base medium formulation in terms of T cell viability on day 3 and T cell expansion on day 6 of cell culture. These components belong to different categories, such as buffer substances, metabolically active components, proteins or trace elements. The diversity of selected components makes it difficult to draw conclusions about potential main effects of single components and synergistic effects of multiple components, so that an experimental design with high resolution was required. We decided on a 2*k* + 1 definitive screening design (Jones and Nachtsheim, 2011) in three levels (in scaled notation, -1, 0, and 1) to investigate main effects, curvature and interactions of the screened components with a minimum number of runs. One experimental block resulted in a total of 26 runs, consisting of 25 formulations and the reference medium with known performance (Supplementary Table 1). T cells isolated from four different donors were investigated in separate experimental blocks, resulting in four randomized complete blocks and a total of 104 runs. T cell viability on day 3 and T cell expansion on day 6 were recorded. The data was processed and served as our training set for the model building process.

**FIGURE 2:**
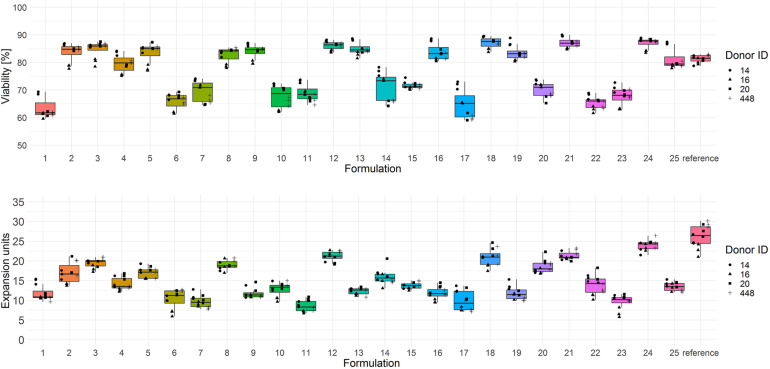
Cryopreserved CD3^+^ T cells purified from human donors (*n* = 4) were thawed and activated using anti-CD3/anti-CD28-coupled magnetic beads in test media or reference media in the presence of IL-7 and IL-15 (10 ng/ml, CellGenix) in three technical replicates. On day 3 cells were labeled with 7-AAD and cell viability was determined (*upper panel*). On day 6 cell counts were determined by flow cytometry and expansion was calculated (*lower panel*). Symbols indicate replicate values of individual donors.

The screening results of T cell viability on day 3 showed two categories of test media with low (<75% viable cells) and high performance (>75% viable cells) (Figure 2, upper panel). In terms of T cell expansion on day 6, each test combination showed a lower performance (median expansion units < 26) than the reference medium (Figure 2, lower panel).

### Data Modeling

To meet the challenge of the high response variability introduced by using cells from different donors, we first built statistical models with the training set of four donors for each experimental block and each response variable separately. Centered and scaled data were used for this step.

First, we defined three different initial model equations (Supplementary Table 2) to account for different possible constellations of main, quadratic and two-way interaction terms of the 12 media components.

Next, we selected three different model algorithms for the statistical modeling: ordinary least squares regression (OLS) with stepwise AIC feature selection, random forest and elastic net regularized general linear models (glmnet) with automated multiple feature selection. To evaluate the model performance on the training set, we performed a tenfold cross-validation and automated hyperparameter tuning during the modeling step. Details of the model hyperparameter tuning of all final models are outlined in Supplementary Table 5.

In this way, nine independent statistical models were generated for each donor and each response variable, resulting in 72 final models. The statistical models were ranked by root mean squared error (RMSE) and coefficient of determination (*R*^2^) and one final model with highest *R*^2^ and lowest RSME was selected for each donor and response variable.

Due to a higher robustness toward overfitting of the data (Zou and Hastie, 2005), we selected best performing glmnet and random forest models over linear regression models for predictions of *in silico* formulations. The eight selected models showed high prediction power on the training set, with *R*^2^ > 0.92 and RMSE less than 1.5 units (Table 1).

**TABLE 1:** Final model and its performance on the train set for each response and each donor.

Donor ID	Response	Model ID	Method	Train RMSE	Train *R*^2^
014	Expansion	b1.model7e	Glmnet	1.16	0.93
016	Expansion	b2.model9e	Glmnet	0.97	0.96
020	Expansion	b3.model8e	Glmnet	1.44	0.92
448	Expansion	b4.model9e	Glmnet	1.06	0.96
014	Viability	b1.model4v	Random forest	1.10	0.98
016	Viability	b2.model9v	Glmnet	0.82	0.99
020	Viability	b3.model9v	Glmnet	1.05	0.99
448	Viability	b4.model9v	Glmnet	1.13	0.99

### Prediction of *in silico* Formulations

Predictions of T cell viability and expansion were carried out via grid search. Component c02 was expensive and sought to be reduced in the final media formulation. Therefore, a constraint of component c02 to medium level was introduced.

We generated 10^5^ random formulations over the experimental space of the 12 screened components and used these as input data for prediction of expansion and viability in the previously selected models for each donor. The formulations were ranked and the top 40 performing formulations for T cell viability or expansion from each of the eight models were pooled, resulting in 320 formulations. These formulations were used for the following clustering step.

### Clustering and Selection of High-Performance *in silico* Formulations

**FIGURE 3:**
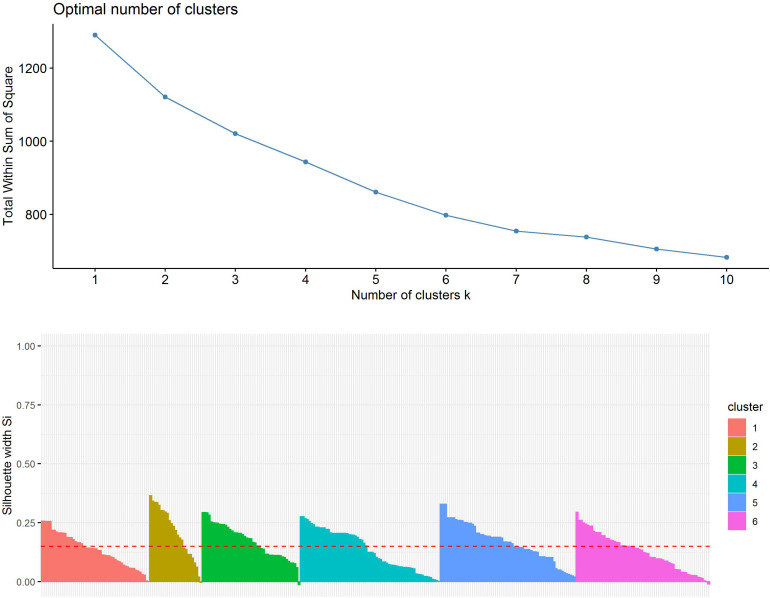
Evaluation of optimal number of clusters by the “elbow” method using the total within Sum of Squares (**upper panel**). Evaluation of cluster integrity by calculation of Silhouette width of each cluster (**lower panel**).

**FIGURE 4:**
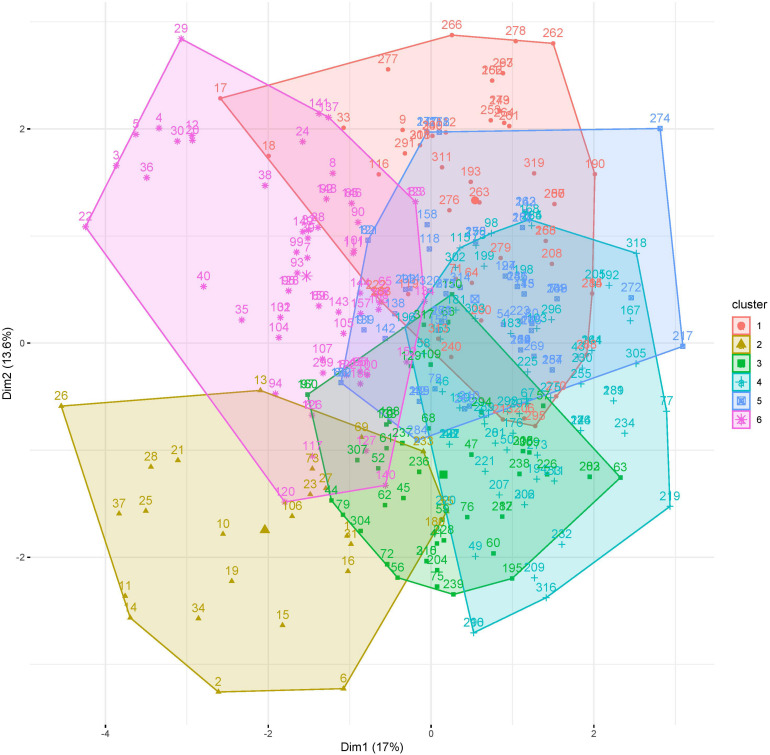
Visualization of clusters 1–6 of 320 media formulations by dimensionality reduction. Dimensions with highest contribution are set on X and Y axis.

First, the clustering tendency of the 320 formulations was assessed by means of the hopkings test (0.423), revealing weak structures. Next, the *k*-means algorithm was used for clustering of the centered and scaled data. To find the optimal number of clusters, the average within-cluster distance to the centroid using the “elbow” method was determined (Figure 3, upper panel). Here we selected six clusters for further analysis. The cluster integrity was examined based on the silhouette width. The average silhouette width was scored 0.15, confirming weak structures (Figure 3, lower panel). For cluster visualization, dimensionality reduction (principal component) was performed and 6 partially overlapping clusters could be identified by plotting the two highest dimensions on X- and Y-axis, exploring 30% of the variation (Figure 4).

While cluster 2 and 6 consisted mainly of formulations of the viability response (92 and 98.44%), formulations in cluster 1 and cluster 4 were mainly from the expansion response (82.69 and 76.12%). Cluster 3 consisted of nearly equal distribution of formulations from viability (53.19%) and expansion response (46.81%), which might explain the overlap on clusters 1 and 4 (Table 2). This data revealed that favorable formulation characteristics differ depending on the response variable, resulting in inhomogeneous clusters.

**TABLE 2:** Number and proportion of media formulations within the clusters.

Cluster number	Response	Formulations (*n*)	Percentage (%)
1	Expansion	43	82.69
	Viability	9	17.31
2	Expansion	2	8.00
	Viability	23	92.00
3	Expansion	22	46.81
	Viability	25	53.19
4	Expansion	51	76.12
	Viability	16	23.88
5	Expansion	41	63.08
	Viability	24	36.92
6	Expansion	1	1.56
	Viability	63	98.44

**FIGURE 5:**
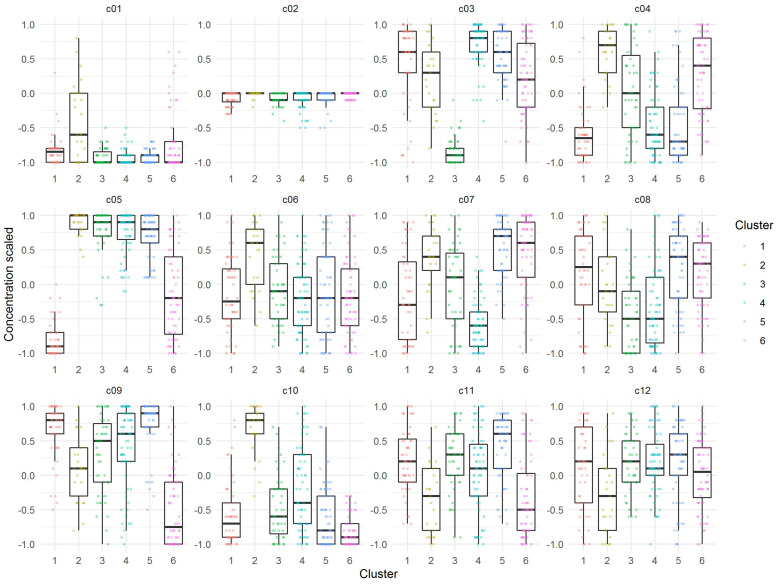
Visualization of relative concentrations of each component in the identified Cluster medium formulations. Dots are representing each individual formulation, while boxes represent the median and spread of the data.

The median of each component of the observations within the six cluster was calculated to obtain a prototypic formulation of each cluster (Figure 5). These cluster medium formulations were “back-evaluated” by predicting the expansion and viability in the selected models for each donor. Data were grouped by cluster and summarized by median of expansion and viability. While the predicted values of viability were very similar for all clusters, the predicted expansion varied markedly (Supplementary Table 3). We therefore selected the medium formulation of cluster 1 and 4 for further experimental evaluation, as both formulations showed higher predicted median values of T cell expansion than the formulations of cluster 2, 3, and 6, respectively. The in-depth evaluation of the clusters revealed that clusters 1, 4, and 5 contained formulations of all donors for the expansion response which correlated with the higher predicted median expansion across all donors. On the contrary, clusters 2, 3, and 6 contained only formulations of three donors (Cluster 3), two donors (Cluster 2) or only one donor (Cluster 6), which in any case correlated with lower predicted median expansion across all donors (Supplementary Figure 1A and Supplementary Table 3). In addition, cluster medium formulation 1 and 4 differed significantly in their characteristics by relative concentrations of component c05 and c08. A weak difference could be identified for components c04, c07, and c010 (Figure 5).

### Evaluation of Selected Cluster Medium Formulations on a New Set of Donors

Cluster medium formulations 1 and 4 were used in a confirmation experiment against six test media obtained by a traditional optimization strategy using a single response *ordinary least squares* regression model that is based on pooled expansion data from the definitive screening design. For the linear regression model, the training data of T cell expansion at day 6 were pooled and a single linear regression model was built to obtain a medium formulation that is characterized by enhanced robustness against donor variability. As expected, the final model performance was lower when compared to the individual models obtained from the machine learning approach (*R*^2^ = 0.85, RMSE = 1.79). Six media formulations from the single response linear regression model that differed in the concentration of the screened components were selected and used for the confirmation experiment (Supplementary Table 4).

To evaluate the performance of the identified media formulation, T cells from a test set of four new donors were expanded in a randomized complete block design, resulting in a total of 36 confirmation runs.

**FIGURE 6:**
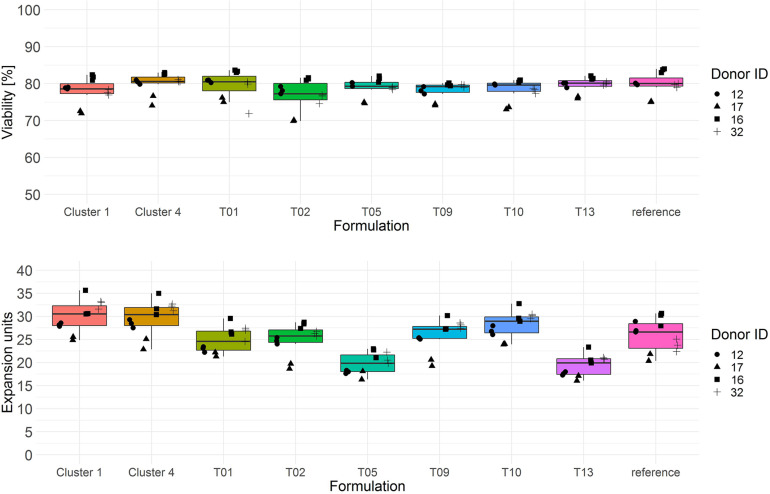
Cryopreserved CD3^+^ T cells purified from human donors (*n* = 4) were thawed and activated using anti-CD3/anti-CD28-coupled magnetic beads in test media or reference media in the presence of IL-7 and IL-15 (10 ng/ml, CellGenix) in three technical replicates. On day 3 cells were labeled with 7-AAD and cell viability was determined **(upper panel)**. On day 6 cell counts were determined by flow cytometry. T cell expansion is expressed in expansion units **(lower panel)**. Symbols indicate replicate values of individual donors.

Both cluster medium formulations showed significantly higher T cell expansion (median > 30 expansion units) compared to all test media from the single response linear regression model as well as to the reference medium (Figure 6, lower panel and Tables 3A,B). T cell viability after culture in cluster medium formulation 1 was significant lower on day 3 in comparison to cluster medium formulation 4 or the reference medium, yet viability was in a very narrow range across all media (>75%) (Figure 6, upper panel).

**TABLE 3:** Comparison of predicted vs. experimental values of the test set for the individual models (median of *n* = 4 models) **(A)** and the single regression model of pooled data **(B)**.

(A)					
**Response**	**Formulation ID**	**Predicted values (median)**	**IQR**	**Experimental values (median)**	**IQR**
Expansion	Cluster 1	30.54	2.16	30.53	4.31
	Cluster 4	31.65	4.87	30.36	3.94
Viability	Cluster 1	85.23	4.31	78.55	2.67
	Cluster 4	85.86	3.94	80.58	1.62

**(B)**					

**Response**	**Formulation ID**	**Predicted values**	**Experimental values (median)**	**IQR**	
Expansion	T01	20.30	24.60	4.17	
	T02	19.99	25.74	2.72	
	T05	19.87	19.86	3.60	
	T09	19.76	27.22	2.59	
	T10	19.74	28.95	3.47	
	T13	19.63	19.90	3.38	

### Evaluation of Model Performance

To reveal the performance of the statistical models, we compared the experimental values for T cell viability and expansion of the test set with the predicted counterparts from the final statistical models of the training set. Using the individual models, the prediction of the T cell expansion averaged over all donors was highly accurate. For cluster medium formulation 1, 30.54 expansion units with an IQR of 2.16 were predicted compared to the experimental value of 30.53 with an IQR of 4.31 expansion units. For cluster medium formulation 4, 31.65 expansion units with an IQR of 4.87 were predicted compared to 30.36 with an IQR of 3.94 experimental expansion units (Table 3A).

The model performance for the viability response on day 3 was less accurate. While the predicted values for both cluster medium formulations were estimated at 85% viability with an IQR of 4.31 and 3.94, the viability in the confirmation experiment was 78.55% with an IQR of 2.67% for Cluster 1 and 80.85% with an IQR of 1.62% for Cluster 4 (Table 3A). The prediction of T cell expansion with the single response regression model for expansion at day 6 was not that accurate compared to the individual models. Four of the six test media formulations showed a significant deviation of the predictions from their experimental values (Table 3B).

To evaluate the benefit of the clustering step, we performed an *in silico* experiment, where only the component-wise median of the best performing formulations from all donor-specific regression models were simulated. This simple ensembling method generated a medium formulation with a predicted expansion of 27.65 units with an IQR of 0.36 and a viability of 83.28% with an IQR of 1.55% averaged over all donors. While the result was very similar in terms of the viability response, the predicted expansion was lower compared to cluster medium formulations 1 and 4, respectively. Taken together our new one-time media optimization approach was able to predict a culture media formulation based on a training set of human donors, that significantly improved expansion of T cells from a new set of human donors in a confirmation experiment.

## Discussion

The biggest challenge in the development of cell culture media for cell therapies is the high variability of biological material obtained from different human donors. This fact complicates a conventional DoE-based sequential optimization strategy consisting of screening, characterization and optimization blocks due to donor-dependent effect sizes of the screened components.

In this work we present the implementation of a machine learning pipeline into DoE-based development of cell culture media, resulting in a one-time media optimization strategy that combines component screening with component level optimization in one experiment by using statistical models that focus on prediction rather than on inference.

The use of competitive modeling algorithms such as random forest or elastic net and cross-validation to assess model performance enabled us to extract extensive statistical information to build individual models for every donor of the training set with improved predictive power. In comparison, predictions made by an interpretable single response regression model based on pooled data of all donors were less accurate due to variability introduced by donor-dependent effect sizes of the screened components.

Instead of aggregating donor data for modeling, which is commonly preferred in a sco strategy when operating with high variance of the data, we pooled high performance predictions from each donor model and used unsupervised clustering to identify formulations with similar characteristics. The formulations in each clusters contained *in silico* formulations from all input models suggesting that a prototypic cluster medium formulation could be beneficial for each donor and would subsequently yield a cell culture medium with high robustness for all donors. This clustering step turned out to be more efficient than a more basal ensembling method that was not supported by unsupervised machine learning. Of note, the small sample size of *n* = 4 donors resulted in weak overlapping cluster structures and a certain risk of under-representation of the population, which could decrease the robustness of the final medium. We evaluated the cluster composition according to the proportion of different donor formulations and selected cluster 1 and 4, which represented all four donors, for the confirmation experiment. Using the median value of each component within the cluster, we were able to confirm a robust expansion reaction for the selected cluster medium formulations in all four donors of the test set.

For the selected cluster media formulations, the predictions for T cell expansion made with the individual donor models precisely matched with the median expansion response observed experimentally for a test set of new donors. Having reliable models that allow predictions of experimental outcomes opens further perspectives for media optimization and characterization. For example, inspection of the mathematical model terms may uncover the contributions of individual media components. One may further modify the formulation by eliminating undesired factors from the formulation *in silico* and re-evaluate manual adjustments of the formulation in the original model. Here, again, component effects that only apply to specific donors can be monitored by back evaluation in the different donor models.

Notably, the predictions for the viability response were less precise than for the expansion response. The spread of the viability over the four donors of the test data set was higher than in the training data set and was likely not in the response space covered by our initial models. This highlights the requirement to use donors in the training data set that reflect the distribution of all observed responses in the donor population as good as possible to improve the predictive power of the models.

Although the viability on day three was not improved in Cluster1 formulation, compared to cluster 4 formulation, both formulations achieved similar T cell expansion on day 6. This suggests that moderate differences in cell viability at the beginning of the culture do not necessarily affect the degree of cell expansion observed later in the culture. Apart from that, a culture medium that confers high cell viability early in culture is always favorable in shorter protocols that rely more on cell viability and substitute exponential expansion of cells by increased starting cell numbers or *in vivo* expansion of cells.

In summary, we have shown that the extension of the traditional DoE-based strategy with a machine learning pipeline allows the generation of statistical models with excellent predictive power for T cell expansion. This new approach might be a competitive alternative to a more traditional strategy in which model interpretation is highly desired in the optimization process. Our pipeline facilitates the discovery of high-performance cell culture media formulations in a one-time optimization approach that achieved high media performance across donors from one training data set without the need for sequential experiments. This applies to primary culture media development as well as to other applications, where responses of individual donors to a specific input vary due to biologic variance, for example in biotechnological process development for T cell therapies with the need to optimize seeding cell density, incubation times, media feed or perfusion regimens, gene transfection procedures etc. The described experimentation strategy can lead to a major reduction of the experimental effort and can shorten development times substantially and thereby help to optimize robust autologous cell products for viable therapies for every particular patient.

## Data Availability Statement

The original contributions presented in the study are included in the article/Supplementary Material, further inquiries can be directed to the corresponding author.

## Author Contributions

PG study conception and design of the experiments, development and coding of the machine learning pipeline, data analysis and interpretation, and drafting of the manuscript. SW study conception, design of experiments, acquisition and interpretation of data, and drafting of the manuscript. Both authors approved the submitted version.

## Conflict of Interest

The authors declare that the research was conducted in the absence of any commercial or financial relationships that could be construed as a potential conflict of interest.
