# Supplementary material for: One-Time Optimization of Advanced T Cell Culture Media Using a Machine Learning Pipeline
Source: Front Bioeng Biotechnol. 2021 Jul 15;9:614324. doi: 10.3389/fbioe.2021.614324 (PMC8320393; doi:10.3389/fbioe.2021.614324)
Supplement: Supplementary file 1 [file Table_1.DOCX]

Supplementary Material

# Supplementary Tables

**Supplementary Table 1.** **Media Formulation Design**

This matrix shows a definitive screening design for 12 components resulting in 25 test formulations. The levels -1 (low), 0 (mid) and 1 (high) represent relative concentrations in scaled units of each component.

| **Formulation ID** | **c01** | **c02** | **c03** | **c04** | **c05** | **c06** | **c07** | **c08** | **c09** | **c10** | **c11** | **c12** |
| --- | --- | --- | --- | --- | --- | --- | --- | --- | --- | --- | --- | --- |
| 1 | 0 | -1 | -1 | 1 | -1 | 1 | -1 | 1 | 1 | 1 | -1 | 1 |
| 2 | 0 | 1 | 1 | -1 | 1 | -1 | 1 | -1 | -1 | -1 | 1 | -1 |
| 3 | -1 | 0 | 1 | 1 | 1 | 1 | 1 | 1 | 1 | -1 | -1 | -1 |
| 4 | 1 | 0 | -1 | -1 | -1 | -1 | -1 | -1 | -1 | 1 | 1 | 1 |
| 5 | 1 | 1 | 0 | -1 | 1 | 1 | -1 | 1 | 1 | -1 | 1 | 1 |
| 6 | -1 | -1 | 0 | 1 | -1 | -1 | 1 | -1 | -1 | 1 | -1 | -1 |
| 7 | 1 | -1 | -1 | 0 | 1 | -1 | 1 | -1 | 1 | -1 | -1 | 1 |
| 8 | -1 | 1 | 1 | 0 | -1 | 1 | -1 | 1 | -1 | 1 | 1 | -1 |
| 9 | 1 | 1 | 1 | 1 | 0 | -1 | 1 | 1 | 1 | 1 | 1 | 1 |
| 10 | -1 | -1 | -1 | -1 | 0 | 1 | -1 | -1 | -1 | -1 | -1 | -1 |
| 11 | 1 | -1 | 1 | -1 | 1 | 0 | 1 | 1 | -1 | 1 | -1 | 1 |
| 12 | -1 | 1 | -1 | 1 | -1 | 0 | -1 | -1 | 1 | -1 | 1 | -1 |
| 13 | 1 | 1 | 1 | 1 | -1 | 1 | 0 | -1 | -1 | -1 | -1 | 1 |
| 14 | -1 | -1 | -1 | -1 | 1 | -1 | 0 | 1 | 1 | 1 | 1 | -1 |
| 15 | -1 | -1 | 1 | 1 | 1 | -1 | -1 | 0 | -1 | -1 | 1 | 1 |
| 16 | 1 | 1 | -1 | -1 | -1 | 1 | 1 | 0 | 1 | 1 | -1 | -1 |
| 17 | 1 | -1 | 1 | 1 | 1 | 1 | -1 | -1 | 0 | 1 | 1 | -1 |
| 18 | -1 | 1 | -1 | -1 | -1 | -1 | 1 | 1 | 0 | -1 | -1 | 1 |
| 19 | 1 | 1 | -1 | 1 | 1 | -1 | -1 | 1 | -1 | 0 | -1 | -1 |
| 20 | -1 | -1 | 1 | -1 | -1 | 1 | 1 | -1 | 1 | 0 | 1 | 1 |
| 21 | -1 | 1 | -1 | 1 | 1 | 1 | 1 | -1 | -1 | 1 | 0 | 1 |
| 22 | 1 | -1 | 1 | -1 | -1 | -1 | -1 | 1 | 1 | -1 | 0 | -1 |
| 23 | 1 | -1 | -1 | 1 | -1 | 1 | 1 | 1 | -1 | -1 | 1 | 0 |
| 24 | -1 | 1 | 1 | -1 | 1 | -1 | -1 | -1 | 1 | 1 | -1 | 0 |
| 25 | 0 | 0 | 0 | 0 | 0 | 0 | 0 | 0 | 0 | 0 | 0 | 0 |

**Supplementary Table 2.**  **Initial model equation**

As starting point for statistical modeling three different model equations are defined per response variable. Automated feature selection towards the final model equation is followed during the modeling process. Model equation are written in R syntax. A) Model equation represents response variable depending on main and two-way interaction terms. B) Model equation represents response variable depending on main, two-way interaction and quadratic terms. C) Model equation represents response variable depending on main and quadratic terms.

**A)**

$$\boldsymbol{response =}{\boldsymbol{(c}\boldsymbol{01+c}\boldsymbol{02+c}\boldsymbol{03+c}\boldsymbol{04+c}\boldsymbol{05+c}\boldsymbol{06+c}\boldsymbol{07+c}\boldsymbol{08+c}\boldsymbol{09+c}\boldsymbol{10+c}\boldsymbol{11+c}\boldsymbol{12)}}^{\boldsymbol{2}}$$

**B)**

$\boldsymbol{response =}{\boldsymbol{(c}\boldsymbol{01+c}\boldsymbol{02+c}\boldsymbol{03+c}\boldsymbol{04+c}\boldsymbol{05+c}\boldsymbol{06+c}\boldsymbol{07+c}\boldsymbol{08+c}\boldsymbol{09+c}\boldsymbol{10+c}\boldsymbol{11+c}\boldsymbol{12)}}^{\boldsymbol{2}}\boldsymbol{+}{\boldsymbol{c}\boldsymbol{01}}^{\boldsymbol{2}}\boldsymbol{+}{\boldsymbol{c}\boldsymbol{02}}^{\boldsymbol{2}}$ **+** ${\boldsymbol{c}\boldsymbol{03}}^{\boldsymbol{2}}$**+** ${\boldsymbol{c}\boldsymbol{04}}^{\boldsymbol{2}}$**+**${\boldsymbol{c}\boldsymbol{05}}^{\boldsymbol{2}}$**+**${\boldsymbol{c}\boldsymbol{06}}^{\boldsymbol{2}}$**+**${\boldsymbol{c}\boldsymbol{07}}^{\boldsymbol{2}}$ **+** ${\boldsymbol{c}\boldsymbol{08}}^{\boldsymbol{2}}$ **+** ${\boldsymbol{c}\boldsymbol{09}}^{\boldsymbol{2}}$ **+** ${\boldsymbol{c}\boldsymbol{10}}^{\boldsymbol{2}}$ **+** ${\boldsymbol{c}\boldsymbol{11}}^{\boldsymbol{2}}\boldsymbol{+}{\boldsymbol{c}\boldsymbol{12}}^{\boldsymbol{2}}$

**C)**

$\boldsymbol{response =c}\boldsymbol{01+c}\boldsymbol{02+c}\boldsymbol{03+c}\boldsymbol{04+c}\boldsymbol{05+c}\boldsymbol{06+c}\boldsymbol{07+c}\boldsymbol{08+c}\boldsymbol{09+c}\boldsymbol{10+c}\boldsymbol{11+c}\boldsymbol{12+}{\boldsymbol{c}\boldsymbol{01}}^{\boldsymbol{2}}\boldsymbol{+}{\boldsymbol{c}\boldsymbol{02}}^{\boldsymbol{2}}$ **+** ${\boldsymbol{c}\boldsymbol{03}}^{\boldsymbol{2}}$**+** ${\boldsymbol{c}\boldsymbol{04}}^{\boldsymbol{2}}$**+**${\boldsymbol{c}\boldsymbol{05}}^{\boldsymbol{2}}$**+**${\boldsymbol{c}\boldsymbol{06}}^{\boldsymbol{2}}$**+**${\boldsymbol{c}\boldsymbol{07}}^{\boldsymbol{2}}$ **+** ${\boldsymbol{c}\boldsymbol{08}}^{\boldsymbol{2}}$ **+** ${\boldsymbol{c}\boldsymbol{09}}^{\boldsymbol{2}}$ **+** ${\boldsymbol{c}\boldsymbol{10}}^{\boldsymbol{2}}$ **+** ${\boldsymbol{c}\boldsymbol{11}}^{\boldsymbol{2}}\boldsymbol{+}{\boldsymbol{c}\boldsymbol{12}}^{\boldsymbol{2}}$

**Supplementary Table 3.**  **Back evaluation of Cluster medium formulation**

Table represents prediction of expansion at day 6 and viability at day 3 for all six cluster medium formulations.

| **Response** | **Formulation ID** | **Predicted values (median)** | **IQR** |
| --- | --- | --- | --- |
| expansion | Cluster 1 | 30.54 | 2.16 |
|  | Cluster 2 | 24.80 | 0.55 |
|  | Cluster 3 | 28.78 | 4.01 |
|  | Cluster 4 | 31.60 | 4.88 |
|  | Cluster 5 | 30.06 | 3.98 |
|  | Cluster 6 | 24.38 | 1.54 |
| viability | Cluster 1 | 85.23 | 1.32 |
|  | Cluster 2 | 81.46 | 2.86 |
|  | Cluster 3 | 84.45 | 3.25 |
|  | Cluster 4 | 85.86 | 1.17 |
|  | Cluster 5 | 86.85 | 1.51 |
|  | Cluster 6 | 86.86 | 4.04 |

**Supplementary Table 4.**  **Final media formulation**

Table represents the relative concentrations in scaled units of each component in the cluster media formulations and six test media formulations used in the confirmation experiment.

| **Formulation ID** | **c01** | **c02** | **c03** | **c04** | **c05** | **c06** | **c07** | **c08** | **c09** | **c10** | **c11** | **c12** |
| --- | --- | --- | --- | --- | --- | --- | --- | --- | --- | --- | --- | --- |
| Cluster 1 | -0.85 | 0 | 0.6 | -0.65 | -0.9 | -0.25 | -0.3 | 0.25 | 0.8 | -0.7 | 0.2 | 0.2 |
| Cluster 4 | -1 | 0 | 0.8 | -0.6 | 0.9 | -0.2 | -0.6 | -0.5 | 0.6 | -0.4 | 0.1 | 0.1 |
| T01 | -1 | 0 | 0.8 | -0.8 | 0.9 | -0.6 | -0.6 | -0.9 | 0.3 | 0.1 | 0.9 | 0.1 |
| T02 | -0.8 | 0 | 0.9 | -0.7 | -0.2 | -0.9 | -0.9 | 0.9 | 0.7 | -1 | 0.9 | -0.8 |
| T05 | -1 | 0 | -1 | -0.5 | 1 | -0.7 | -0.6 | -1 | -0.3 | -0.8 | 0.9 | 0.8 |
| T09 | -1 | 0 | 0.6 | -0.3 | 1 | 0.4 | -0.1 | 0.9 | 0.9 | -0.5 | 0.7 | -0.8 |
| T10 | -1 | -0.1 | 0.6 | -1 | 0.9 | -1 | -0.5 | 0.2 | 0.9 | 0.6 | 0.3 | 0.1 |
| T13 | -1 | -0.3 | 1 | -0.7 | 0 | -0.8 | -1 | -1 | 0.8 | -0.8 | -0.8 | 0.5 |

**Supplementary Figure 1.**

Pie charts represent the composition of each cluster for expansion response (A) and viability response (B). Colors represent the four different donors and the numbers next to each pie chart indicate the number of formulations from the respective donor model in that cluster.

A


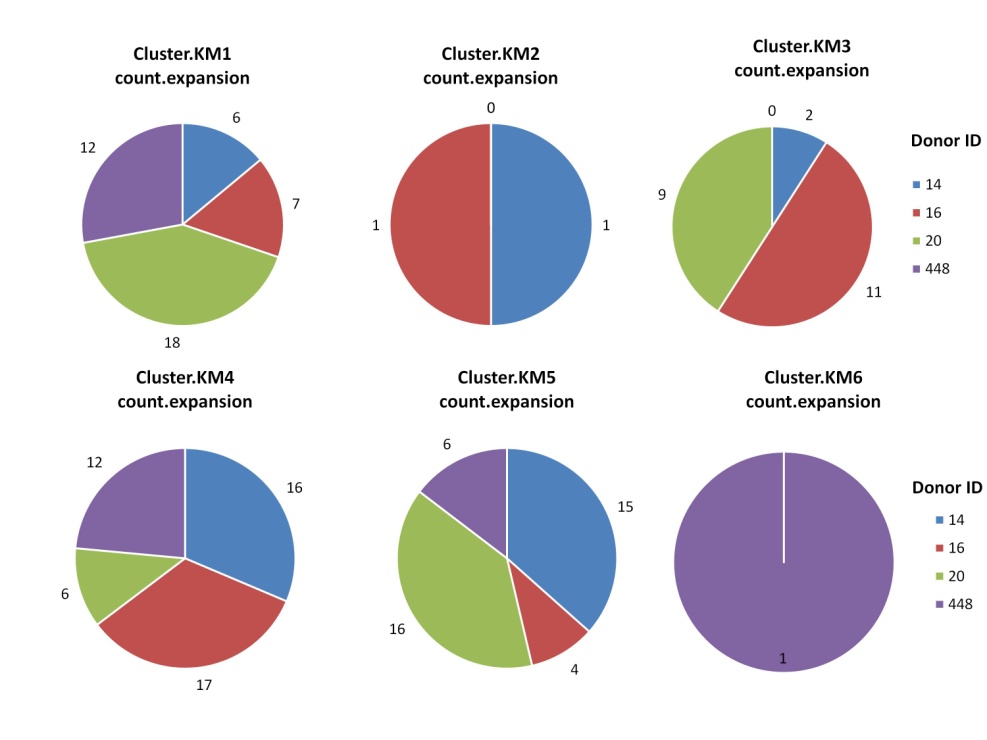


B


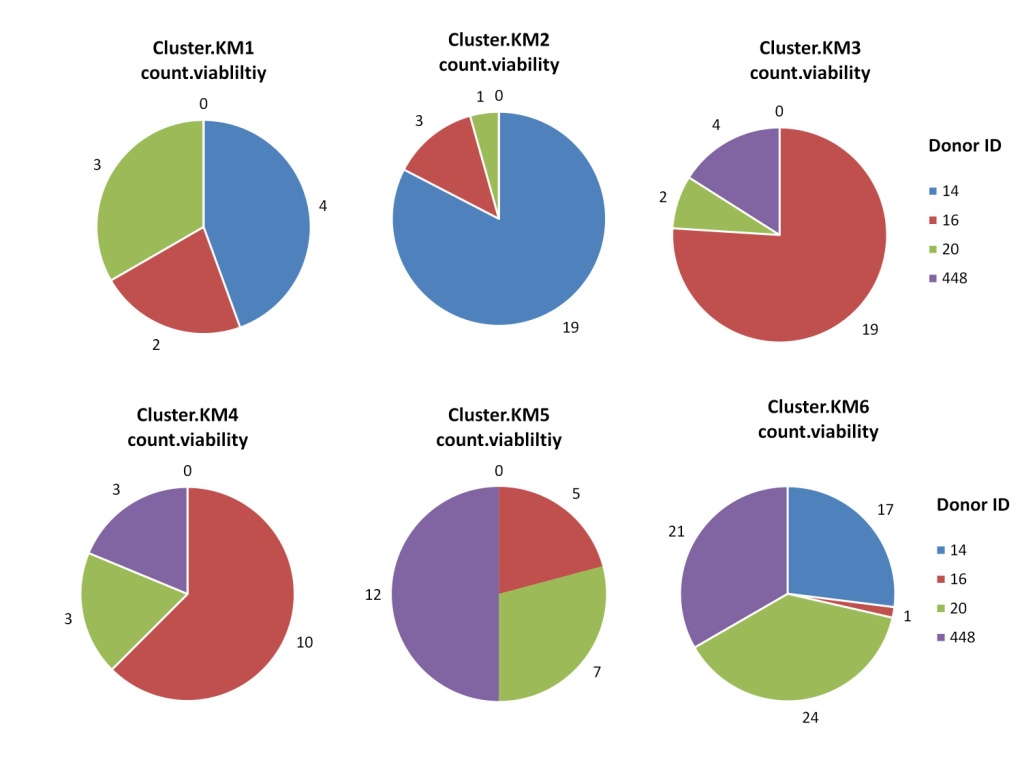


**Supplementary Table 5.**  **Model summary of hyper parameter tuning**

Table represents summary of hyper parameter tuning of each final individual donor model and the linear regression model of the aggregated donor data. Glmnet model hyper parameter alpha was set at alpha 0.10, 0.55. Lambda is automatically generated in the range of 0 to lambda.max which is the smallest value for lambda such that all the coefficients are zero. For alpha=0(ridge).

For random forest default maximum of mtry hyper parameter is the (rounded down) square root of the number of variables. Minimum node size is 5 and splitrules are variance or extratrees.

OLS is performed with stepwise selection AIC criterion.

**d6.b1.model7**

**
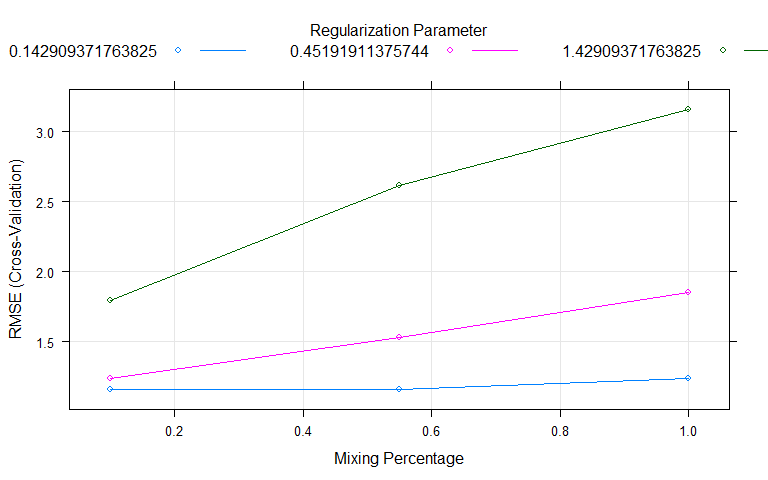
**

Model: glmnet

Resampling: Cross-Validated (10 fold)

Resampling results across tuning parameters:

alpha lambda RMSE Rsquared MAE

0.10 0.1429094 1.157387 0.9281256 0.9843731

0.10 0.4519191 1.233941 0.9264087 1.0576106

0.10 1.4290937 1.795971 0.9048248 1.5614917

0.55 0.1429094 1.156766 0.9300615 0.9809340

0.55 0.4519191 1.533247 0.9001605 1.3342673

0.55 1.4290937 2.613493 0.7705236 2.2371058

1.00 0.1429094 1.239063 0.9231909 1.0665165

1.00 0.4519191 1.854353 0.8519116 1.5764644

1.00 1.4290937 3.162744 0.6250251 2.7209914

RMSE was used to select the optimal model using the smallest value.

The final values used for the model were alpha = 0.55 and lambda = 0.1429094.

**d6.b2.model9**

**
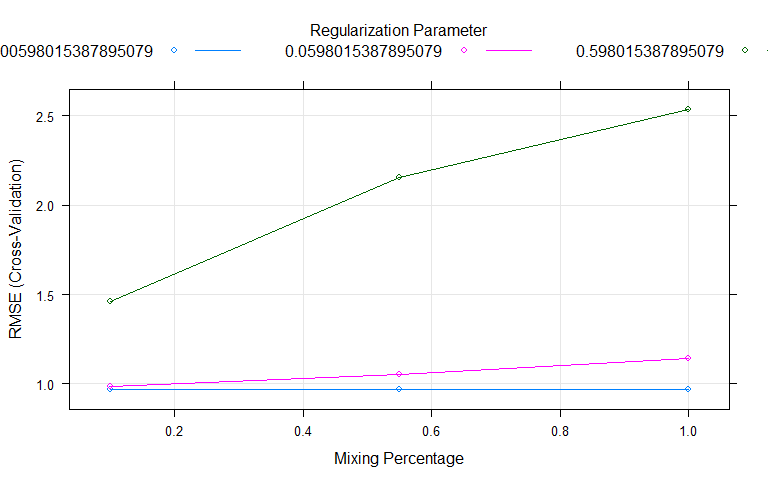
**

Model: glmnet

Resampling: Cross-Validated (10 fold)

Resampling results across tuning parameters:

alpha lambda RMSE Rsquared MAE

0.10 0.005980154 0.9676725 0.9647495 0.7505733

0.10 0.059801539 0.9873984 0.9644409 0.7615270

0.10 0.598015388 1.4606754 0.9511158 1.1830515

0.55 0.005980154 0.9685449 0.9645661 0.7512109

0.55 0.059801539 1.0542209 0.9615996 0.8063434

0.55 0.598015388 2.1536242 0.8446272 1.7777865

1.00 0.005980154 0.9705280 0.9645009 0.7526999

1.00 0.059801539 1.1417719 0.9563227 0.8842936

1.00 0.598015388 2.5382767 0.7631358 2.1201481

RMSE was used to select the optimal model using the smallest value.

The final values used for the model were alpha = 0.1 and lambda = 0.005980154.

glmnet

**d6.b3.model8**

**
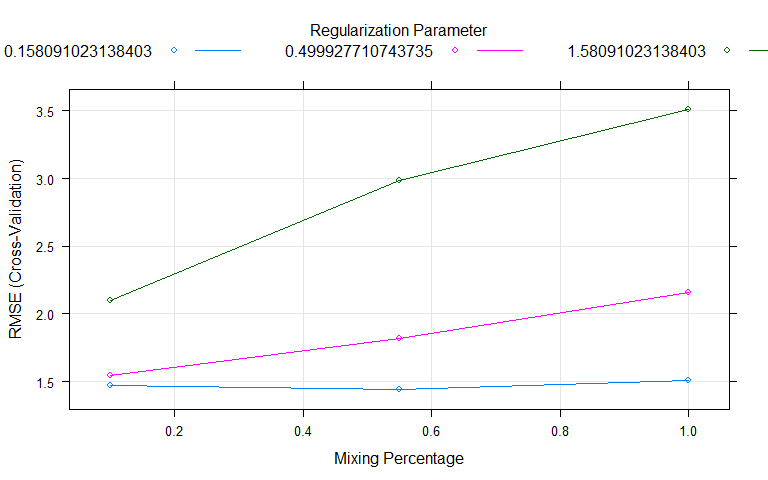
**

Model: glmnet

Resampling: Cross-Validated (10 fold)

Resampling results across tuning parameters:

alpha lambda RMSE Rsquared MAE

0.10 0.1580910 1.469663 0.9151372 1.213302

0.10 0.4999277 1.542820 0.9165851 1.260309

0.10 1.5809102 2.098907 0.9060780 1.772185

0.55 0.1580910 1.439666 0.9221912 1.182678

0.55 0.4999277 1.821437 0.8954202 1.540092

0.55 1.5809102 2.983640 0.7038051 2.618049

1.00 0.1580910 1.505089 0.9175508 1.234208

1.00 0.4999277 2.158002 0.8308852 1.861299

1.00 1.5809102 3.513142 0.5762208 3.108718

RMSE was used to select the optimal model using the smallest value.

The final values used for the model were alpha = 0.55 and lambda = 0.158091.

glmnet

**d6.b4.model9**

**
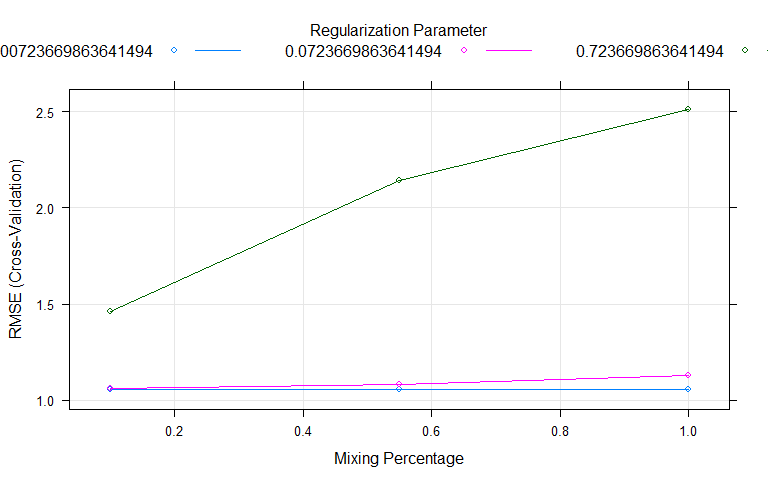
**

Model: glmnet

Resampling: Cross-Validated (10 fold)

Resampling results across tuning parameters:

alpha lambda RMSE Rsquared MAE

0.10 0.007236699 1.056832 0.9619937 0.8240233

0.10 0.072366986 1.061346 0.9616997 0.8323796

0.10 0.723669864 1.460577 0.9516933 1.2365963

0.55 0.007236699 1.055826 0.9619903 0.8226140

0.55 0.072366986 1.080666 0.9598859 0.8626029

0.55 0.723669864 2.141684 0.8688437 1.8922578

1.00 0.007236699 1.055948 0.9619447 0.8233037

1.00 0.072366986 1.130043 0.9559587 0.9154399

1.00 0.723669864 2.512226 0.8002636 2.2755593

RMSE was used to select the optimal model using the smallest value.

The final values used for the model were alpha = 0.55 and lambda = 0.007236699.

**d3.b1.model4v**


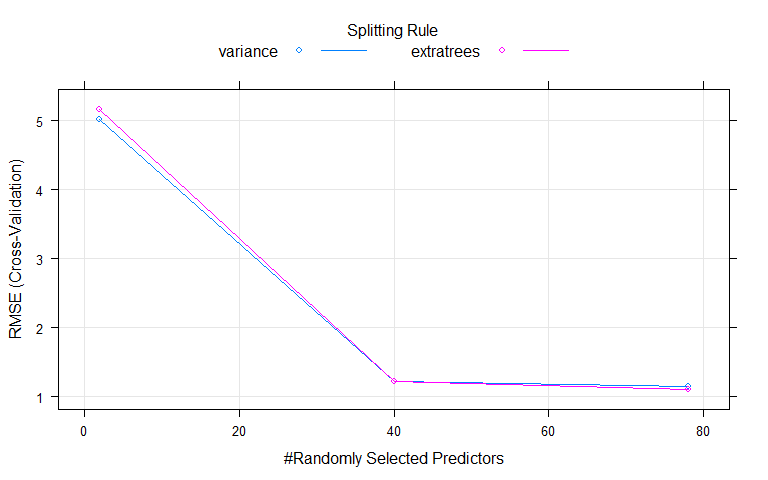


Model: Random Forest

Resampling: Cross-Validated (10 fold)

Resampling results across tuning parameters:

mtry splitrule RMSE Rsquared MAE

2 variance 5.012510 0.8377748 4.6375234

2 extratrees 5.166180 0.8236688 4.7751467

40 variance 1.213532 0.9835203 0.9787950

40 extratrees 1.222774 0.9843204 0.9839751

78 variance 1.151109 0.9832227 0.9018796

78 extratrees 1.100065 0.9846170 0.8555405

Tuning parameter 'min.node.size' was held constant at a value of 5

RMSE was used to select the optimal model using the smallest value.

The final values used for the model were mtry = 78, splitrule = extratrees and

min.node.size = 5.

**d3.b2.model9v**


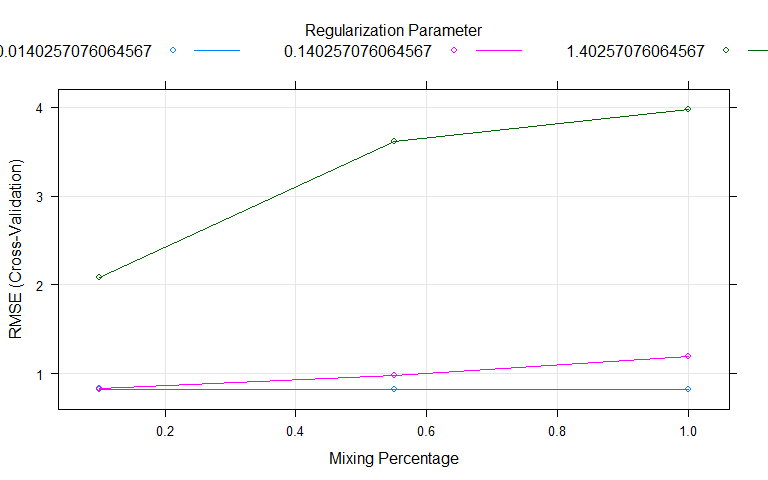


Model: glmnet

Resampling: Cross-Validated (10 fold)

Resampling results across tuning parameters:

alpha lambda RMSE Rsquared MAE

0.10 0.01402571 0.8188770 0.9913559 0.6857669

0.10 0.14025708 0.8324077 0.9911109 0.7066029

0.10 1.40257076 2.0793684 0.9792512 1.6954300

0.55 0.01402571 0.8212156 0.9912503 0.6862672

0.55 0.14025708 0.9803416 0.9872317 0.8251115

0.55 1.40257076 3.6135525 0.8430558 3.1196951

1.00 0.01402571 0.8206999 0.9911524 0.6869484

1.00 0.14025708 1.1970891 0.9813527 1.0116925

1.00 1.40257076 3.9804054 0.7990298 3.4266460

RMSE was used to select the optimal model using the smallest value.

The final values used for the model were alpha = 0.1 and lambda = 0.01402571.

**d3.b3.model9v**


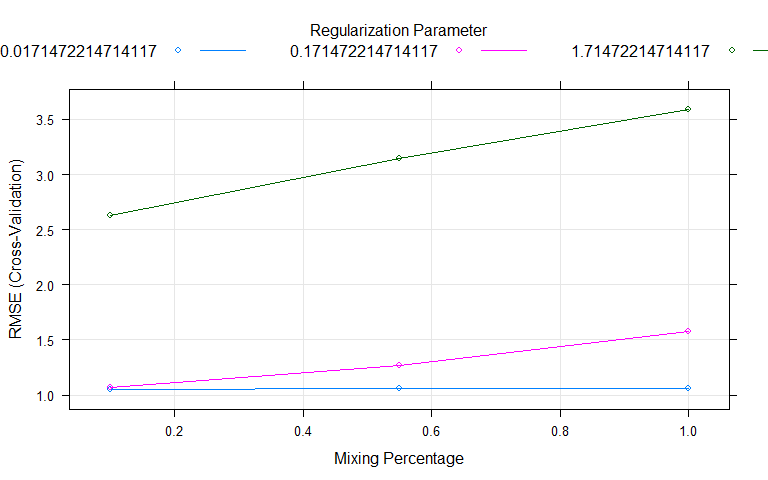


Model: glmnet

Resampling: Cross-Validated (10 fold)

Resampling results across tuning parameters:

alpha lambda RMSE Rsquared MAE

0.10 0.01714722 1.049349 0.9907045 0.8075767

0.10 0.17147221 1.069456 0.9906550 0.8319522

0.10 1.71472215 2.631751 0.9766337 2.0507273

0.55 0.01714722 1.056843 0.9906306 0.8118276

0.55 0.17147221 1.264906 0.9864776 0.9595172

0.55 1.71472215 3.147799 0.9290161 2.3246190

1.00 0.01714722 1.062362 0.9905346 0.8173782

1.00 0.17147221 1.576473 0.9783003 1.2286425

1.00 1.71472215 3.593348 0.8944772 2.7393744

RMSE was used to select the optimal model using the smallest value.

The final values used for the model were alpha = 0.1 and lambda = 0.01714722.

**d3.b4.model9v**


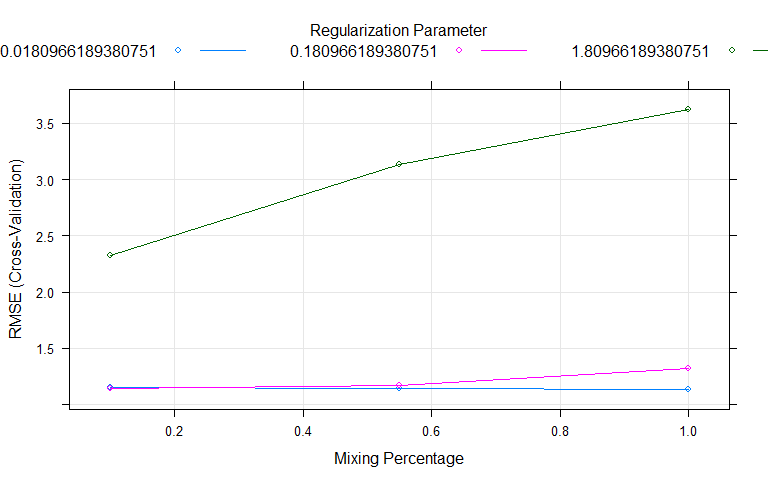


Model: glmnet

Resampling: Cross-Validated (10 fold)

Resampling results across tuning parameters:

alpha lambda RMSE Rsquared MAE

0.10 0.01809662 1.147614 0.9899444 0.9089653

0.10 0.18096619 1.146321 0.9899854 0.8993402

0.10 1.80966189 2.328356 0.9826614 2.0283909

0.55 0.01809662 1.139840 0.9899432 0.9023430

0.55 0.18096619 1.171595 0.9877214 0.9332249

0.55 1.80966189 3.135080 0.9345157 2.4487334

1.00 0.01809662 1.132164 0.9899357 0.8952389

1.00 0.18096619 1.322462 0.9838247 1.0598284

1.00 1.80966189 3.627563 0.9011724 2.8768152

RMSE was used to select the optimal model using the smallest value.

The final values used for the model were alpha = 1 and lambda = 0.01809662.

**d6.model.1e (all Donors aggregated)**

Linear Regression with Stepwise Selection

Resampling: Cross-Validated (10 fold)

Resampling results:

RMSE Rsquared MAE

1.795758 0.8528167 1.443513

Coefficients:

Estimate Std. Error t value Pr(>|t|)

(Intercept) 13.4971 0.4862 27.759 < 2e-16 ***

A -3.1292 0.1071 -29.216 < 2e-16 ***

B 2.4203 0.1054 22.971 < 2e-16 ***

C 0.1710 0.1091 1.566 0.118425

D -0.8329 0.1092 -7.629 4.19e-13 ***

E 0.2156 0.1090 1.978 0.048920 *

F 0.2114 0.1093 1.935 0.054093 .

G -0.4587 0.1095 -4.190 3.79e-05 ***

I 0.9545 0.1074 8.890 < 2e-16 ***

J -0.4237 0.1054 -4.022 7.52e-05 ***

K 0.5355 0.1094 4.893 1.72e-06 ***

L 0.5068 0.1092 4.642 5.42e-06 ***

AB -4.9271 1.4695 -3.353 0.000916 ***

AC -2.5227 0.8824 -2.859 0.004584 **

AD 3.5097 1.3204 2.658 0.008333 **

AE -3.2348 0.9936 -3.255 0.001278 **

AF 2.3111 1.0086 2.291 0.022722 *

AG -6.4593 2.2254 -2.902 0.004011 **

AH 3.6892 1.3471 2.739 0.006583 **

AI 3.1862 1.4184 2.246 0.025498 *

AJ 3.7758 1.6119 2.342 0.019890 *

AK -4.3838 1.7619 -2.488 0.013456 *

AL 4.3948 1.6439 2.673 0.007970 **

BC 0.8676 0.6000 1.446 0.149341

Signif. codes: 0 ‘***’ 0.001 ‘**’ 0.01 ‘*’ 0.05 ‘.’ 0.1 ‘ ’ 1
